# Immunogenicity and plasmid delivery pathways of non-invasive *Lactococcus lactis*-vectored mucosal DNA vaccination

**DOI:** 10.1128/iai.00460-25

**Published:** 2025-11-28

**Authors:** Sarana Kawashima, Keita Takahashi, Daiki Yanagisawa, Chitose Irikura, Hiroki Kondo, Naoki Inoue, Juri Koizumi, Tetsuo Koshizuka

**Affiliations:** 1 Laboratory of Microbiology and Immunology, Gifu Pharmaceutical University 12784, Gifu, Japan; Tsinghua University, Beijing, China

**Keywords:** *Lactococcus lactis*, DNA vaccine, mucosal vaccine, phagocytosis, bactofection

## Abstract

Mucosal DNA vaccination using a non-invasive *Lactococcus lactis* (LL) vector has been investigated. However, its immunogenicity and plasmid transfer mechanisms remain largely unknown. In this study, we investigated the intranasal delivery of LL carrying a mammalian enhanced green fluorescent protein (EGFP)-expressing plasmid and the cellular pathways underlying DNA transfer. Intranasally administered LL was primarily localized on the nasal epithelial surfaces, and a smaller fraction penetrated the subepithelial tissues. Intranasal administration of LL-carrying pLEC-EGFP plasmid induces antigen-specific serum IgG and mucosal IgA responses. *In vitro* co-culture analyses demonstrated that plasmid delivery and expression occurred in phagocytic cell lines but not in epithelial cell lines. This transfer was inhibited by compounds specific for phagocytosis, consistent with the observed time course of DNA transfer and localization of LL within Lamp-1^+^ phagolysosomes. In contrast, compounds for bactericidal mechanisms, including lysosomal acidification, reactive oxygen species, and reactive nitrogen species, did not affect DNA transfer. As our findings suggest that phagocytosis is the primary pathway for plasmid delivery by non-invasive LL vectors in cell culture assays, further studies to confirm these findings in animal models are warranted to develop new strategies for improved LL-based mucosal DNA vaccines.

## INTRODUCTION

DNA vaccines emerged decades ago as a promising approach in the field of immunization, offering several advantages, including high stability, ease of large-scale production, and the potential for rapid development, over traditional vaccine strategies or even novel genetic vaccine platforms such as mRNA or viral vectors (1, 2). Usually, DNA vaccines consist of plasmid DNA encoding antigenic proteins that lead to the production of target antigens in host cells and subsequent immune responses. Although DNA vaccines against COVID-19 have been put into practical use in humans (3, 4), further improvements in gene delivery efficiency and immunogenicity are required for the application of DNA vaccine platforms to other infectious diseases or cancers (2).

Mucosal immunity serves as the first line of defense against pathogens at their entry sites and can also stimulate systemic immune responses, offering a comprehensive protective strategy (5). However, conventional DNA vaccine delivery methods are not suitable for targeting mucosal tissues (2). To address this limitation, several bacterial species have been explored as delivery vehicles that are capable of invading host cells, releasing plasmid DNA, and eliciting immune responses. These include attenuated strains of *Shigella*, *Salmonella*, and *Listeria,* as well as genetically modified *Lactococcus lactis* (LL) engineered to invade epithelial cells (6–16).

Alternatively, the use of non-invasive bacteria as delivery vehicles for DNA vaccines presents potential safety advantages over invasive species. Among these, nonpathogenic *Escherichia coli* and LL have been frequently used for mucosal applications (17–20). LL is well known for its probiotic properties, has a long-standing record of safe use in food production, and is classified as generally recognized as safe. Moreover, LL can modulate immune responses through microbe-associated molecular patterns (6). However, the mechanisms of plasmid DNA delivery to host cells and the subsequent induction of immunogenic responses by LL-engineered DNA vaccines remain largely unknown.

We previously reported the successful delivery of a mammalian enhanced green fluorescent protein (EGFP)-expressing plasmid (pLEC-EGFP) to enterocytes from orally administered LL carrying the plasmid (LL/pLEC-EGFP) (21). Based on this study, we assessed the immunogenicity of LL/pLEC-EGFP following mucosal administration and found that intranasal administration induced antigen-specific antibody responses. To further clarify the basis of this response, we investigated the cellular mechanisms underlying plasmid delivery from LL to host cells in nasal tissues.

## RESULTS

### Immunogenicity of LL/pLEC-EGFP via different administration routes

**Fig 1:**
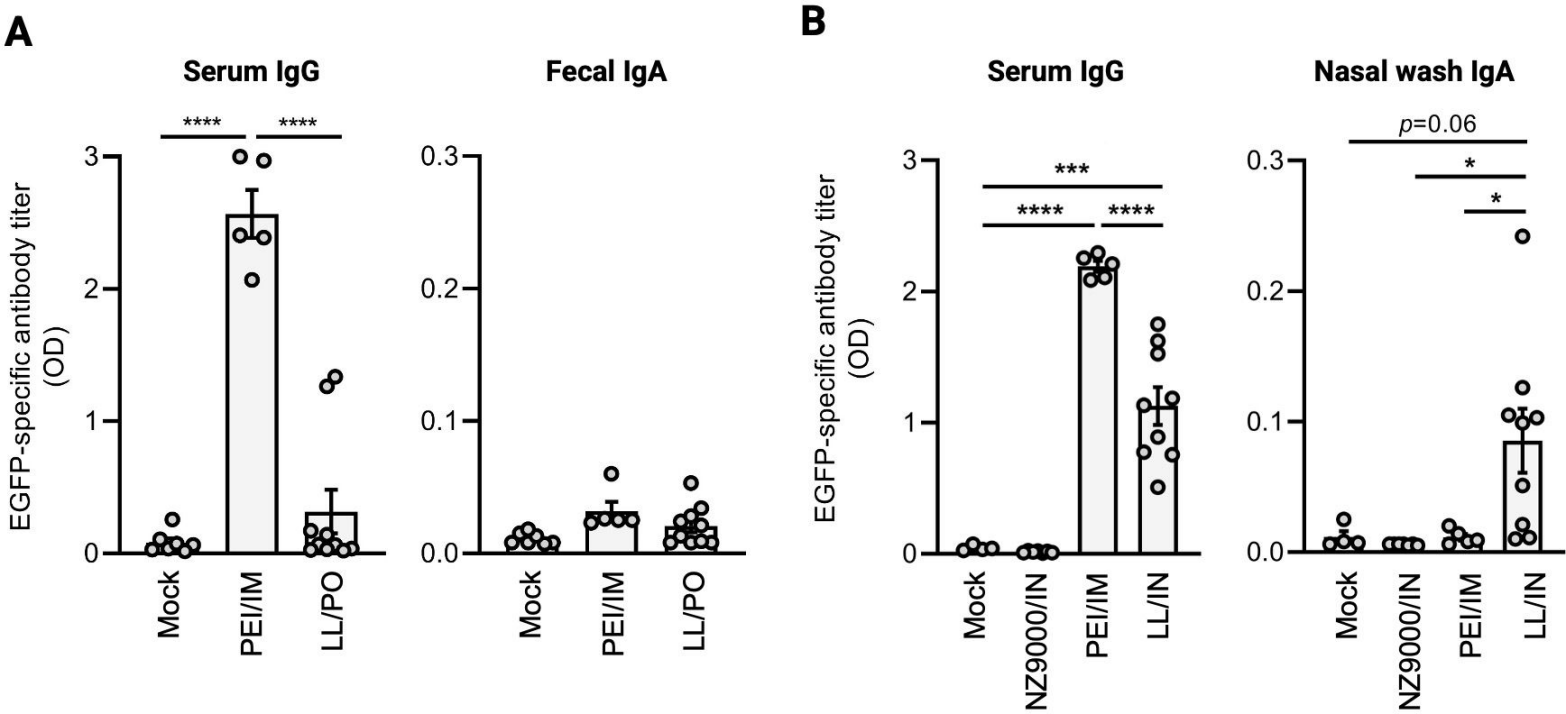
Evaluation of the immunogenicity of LL-vectored oral or nasal DNA vaccines. (**A**) EGFP-specific serum IgG and fecal IgA in mice orally administered with phosphate-buffered saline (PBS) (mock, *n* = 7) or LL/pLEC-EGFP (LL/PO, *n* = 10) or intramuscularly administered with purified pLEC-EGFP plasmid with *in vivo*-jetPEI (PEI/IM, *n* = 5). Data are expressed as mean ± SEM, with individual mouse data points. (**B**) EGFP-specific serum IgG and nasal wash IgA in mice nasally administered with PBS (mock, *n* = 4), non-genetically modified LL (NZ9000/IN, *n* = 7; some data points are behind other points), LL/pLEC-EGFP (LL/IN, *n* = 9), or intramuscularly administered with purified pLEC-EGFP plasmid with *in vivo*-jetPEI (PEI/IM, *n* = 5). Data are expressed as mean ± SEM, with individual mouse data points. *P < 0.05, ***P < 0.001, ****P < 0.0001, by one-way ANOVA with Tukey’s test.

To assess the immunogenicity of the LL-vectored DNA vaccine administered via the mucosal route, mice received 2 × 10^9^ CFU of LL/pLEC-EGFP via oral (LL/PO) or intranasal (LL/IN) administration. As a benchmark and negative control, the mice received intramuscular injections of the pLEC-EGFP plasmid complexed with *in vivo*-jetPEI (PEI/IM) or intranasal administration of the parental LL strain NZ9000 lacking the plasmid (NZ9000/IN), respectively. EGFP-specific serum IgG titers were significantly elevated in LL/IN and PEI/IM mice but not in LL/PO and NZ9000/IN mice (Fig. 1). EGFP-specific mucosal IgA was detected only in the LL/IN group (Fig. 1). Based on an estimated plasmid copy number of 45–85 per LL (22) and the plasmid size of 7.4 kb, the effective plasmid dose delivered by intranasal LL/pLEC-EGFP was approximately 1 µg. Thus, direct comparison between LL/IN and PEI/IM groups should be made with caution. As the pLEC-EGFP plasmid contains an intron and an EGFP-coding sequence downstream of the cytomegalovirus (CMV) promoter (Fig. S1A), and EGFP expression in LL/pLEC-EGFP was not detected by Western blotting (Fig. S1B), these results suggest that nasal administration of LL/pLEC-EGFP delivered plasmid DNA to host cells, leading to EGFP expression and induction of antigen-specific antibody responses.

### Localization of nasally administered LL in the nasal cavity

**Fig 2:**
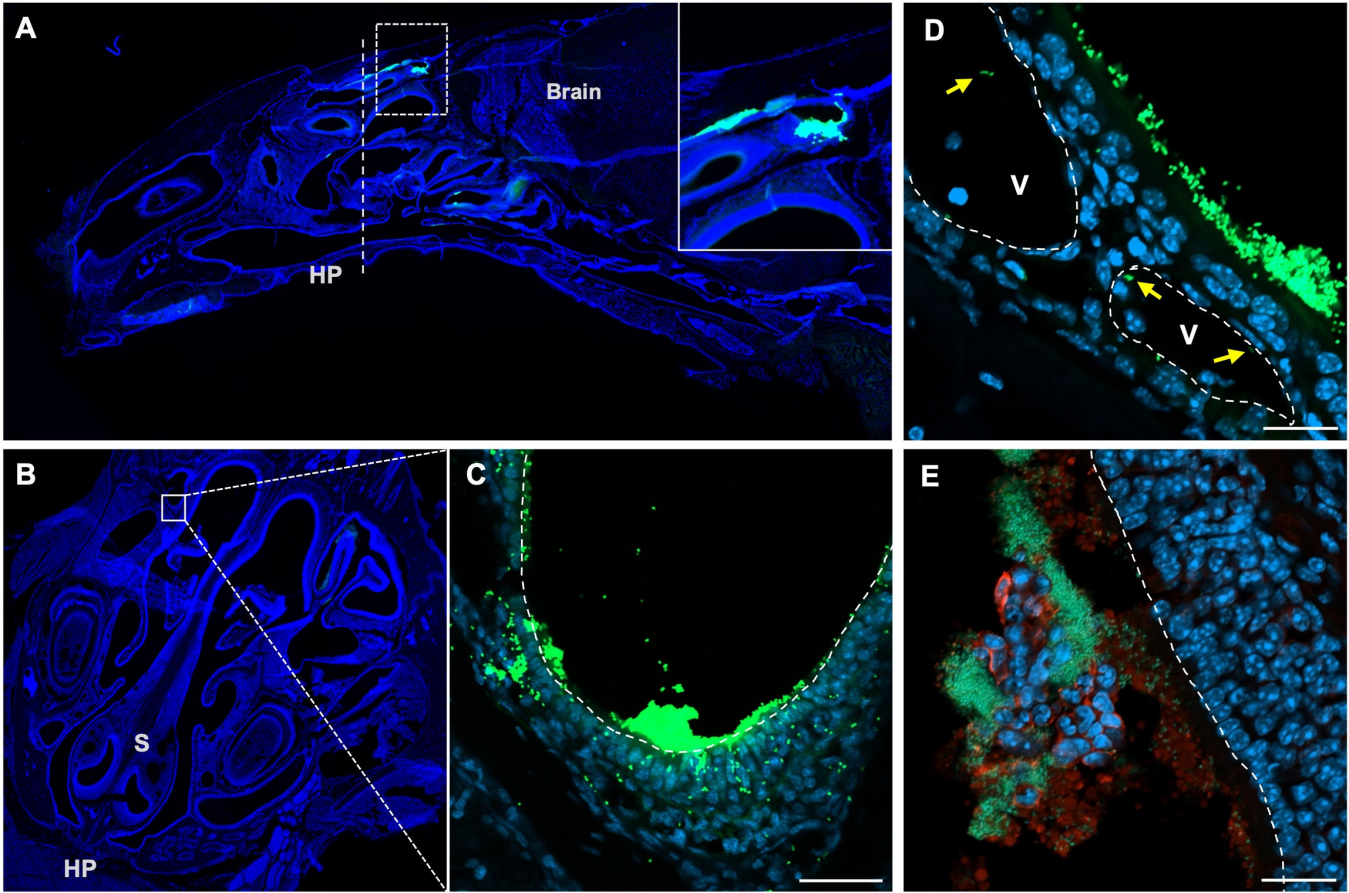
Localization of intranasally administered LL. Mice were intranasally administered 2 × 10^9^ CFU of CFSE-labeled LL (green). Four hours after administration, the nasal tissues were excised, decalcified, and frozen sections were prepared. The sections were stained with Hoechst 33342 (blue). Whole structures of longitudinal (**A**) and cross-sections (**B**) obtained using fluorescence microscopy. The dashed line in panel **A** represents the cross-sectional plane. The dashed box in panel **A** indicates the region magnified in the inset. HP, hard palate; S, septum. (**C**) Confocal microscopy image corresponding to the area indicated by the white box in panel **B**. Dashed line indicates the epithelial surface. (**D**) Confocal microscopic image of a separate nasal tissue section showing occasional CFSE-LL localization within blood vessels. V, blood vessels. Dashed lines indicate blood vessel walls. Yellow arrows indicate CFSE-LL within the blood vessels. (**E**) Confocal images of nasal tissue sections stained with the anti-Ly6G antibody (red) and Hoechst 33342 (blue). Dashed line indicates the epithelial surface. Scale bars indicate 20 µm.

To investigate the mechanism of antigen-specific immune responses in LL/IN, 2 × 10^9^ CFU of carboxyfluorescein succinimidyl ester (CFSE)-labeled LL (CFSE-LL) was nasally administered, and its localization in the nasal cavity was tracked 4 h post-inoculation. Fluorescence microscopy showed that CFSE-LL predominantly adhered to epithelial surfaces, especially on the dorsal side (Fig. 2A). Fewer CFSE-LLs were observed within the subepithelial tissues and blood vessels (Fig. 2B through D). Some slices were co-stained with anti-Ly6G antibody, and some CFSE-LLs were surrounded by Ly6G^+^ neutrophils in the lumen (Fig. 2E; Fig. S2). CFSE-LL signals were also observed colocalized with Ly6G^+^ regions lacking nuclei (Fig. 2E; Fig. S2), suggesting that these bacteria may be trapped within NET-like structures formed in the nasal lumen. These results suggest that epithelial cells, luminal neutrophils, subepithelial cells, and blood cells are potential plasmid recipients from LL. We attempted to identify potential cell types interacting with CFSE-LL by immunofluorescence staining for CD11b, CD11c, and Siglec-F in nasal tissue sections. However, no specific staining was obtained, likely due to epitope loss during the decalcification process required for sample preparation. To identify recipient cells, we also attempted to detect EGFP-expressing cells in the nasal tissues; however, no EGFP-positive cells were observed (Fig. S3), probably because of limited transgene expression or restricted localization.

### *In vitro* analysis of target cell types receiving plasmid DNA from LL

**Fig 3:**
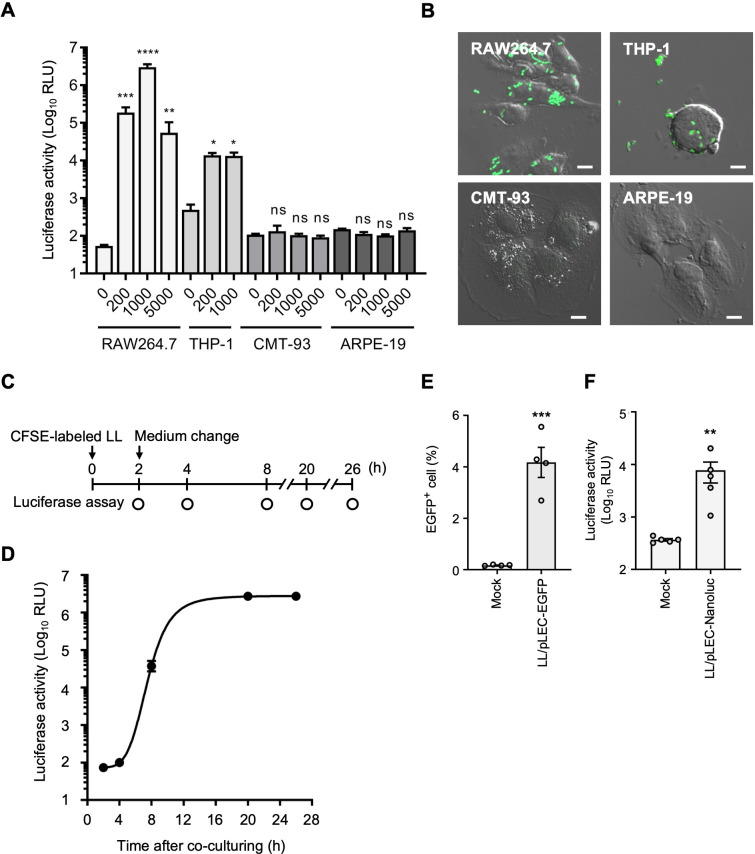
Plasmid DNA delivery from LL to different cell types. (**A**) Luciferase activity in RAW264.7, THP-1, CMT-93, and ARPE-19 cells co-cultured with LL/pLEC-Nanoluc at the indicated cell-to-bacteria ratios for 20 h. Data are mean ± SEM (*n* = 4). *P < 0.05, **P < 0.01, ***P < 0.001, ****P < 0.0001, ns, not significant by one-way ANOVA with Dunnett’s test (vs ratio 0 for each cell line). (**B**) Fluorescent and phase-contrast images of cells co-cultured with CFSE-labeled LL for 4 h. (**C**) Schematic of time-course experiments. RAW264.7 cells were co-cultured with LL/pLEC-Nanoluc or CFSE-labeled LL; after 2 h, medium was replaced with gentamicin-containing medium, and luciferase activity was measured at the indicated times. (**D**) Time course of luciferase activity in RAW264.7 cells (mean ± SEM, *n* = 5/time point; the symbols hide some error bars). (**E**) Representative percentage of EGFP-expressing RAW264.7 cells after 20 h co-culture with LL/pLEC-EGFP (mean ± SEM, *n* = 4). ***P < 0.001 by Student’s *t*-test. (**F**) Luciferase activity in adhered splenocytes co-cultured with LL/pLEC-Nanoluc for 24 h (mean ± SEM, *n* = 5). **P < 0.01 by Mann-Whitney U-test. Cell-to-bacteria ratio was 1:1,000 for panels B, D, and E and 1:10 for panel F.

As a couple of attempts to identify cell types *in vivo* faced technical difficulties, we decided to focus on *in vitro* experiments comparing the plasmid delivery efficiency between epithelial cells (CMT-93 and ARPE-19) and phagocytic cells (RAW264.7 and THP-1). Co-culture of LL/pLEC-Nanoluc with CMT-93 and ARPE-19 cells resulted in luciferase expression at background levels, whereas significant expression was observed in RAW264.7 and THP-1 cells (Fig. 3A).

Consistent with the selective luciferase expression in these phagocytic but not in epithelial cell lines, internalization of CFSE-LL was detected in RAW264.7 and THP-1 cells, but not in CMT-93 or ARPE-19 cells after 4 h of co-culture (Fig. 3B). To characterize plasmid transfer in RAW264.7, we performed a time-course analysis, flow cytometry, and immunofluorescence. Time-course analysis revealed that luciferase activity in RAW264.7 cells was detectable at 8 h, reached a plateau at 20 h, and was sustained for at least 26 h (Fig. 3C and D). Flow cytometry analysis showed that 4% of RAW264.7 cells expressed detectable levels of EGFP following co-culture with LL/pLEC-EGFP (Fig. 3E; Fig. S4). The EGFP expression in RAW264.7 cells was also confirmed by immunofluorescence analysis (Fig. S5). To further validate plasmid delivery in primary immune cells, freshly isolated mouse splenocytes were co-cultured with LL/pLEC-Nanoluc. After removal of non-adherent splenocytes and bacteria, adherent splenocytes, mainly composed of macrophages (23, 24), were incubated for 24 h. Luciferase activity was detected in these cells but not in the mock control (Fig. 2F). These results indicate that LL can deliver plasmid DNA to primary phagocytic cells, consistent with our observations in macrophage-like cell lines.

### Determination of the pathway for the internalization of LL by phagocytic cells

**Fig 4:**
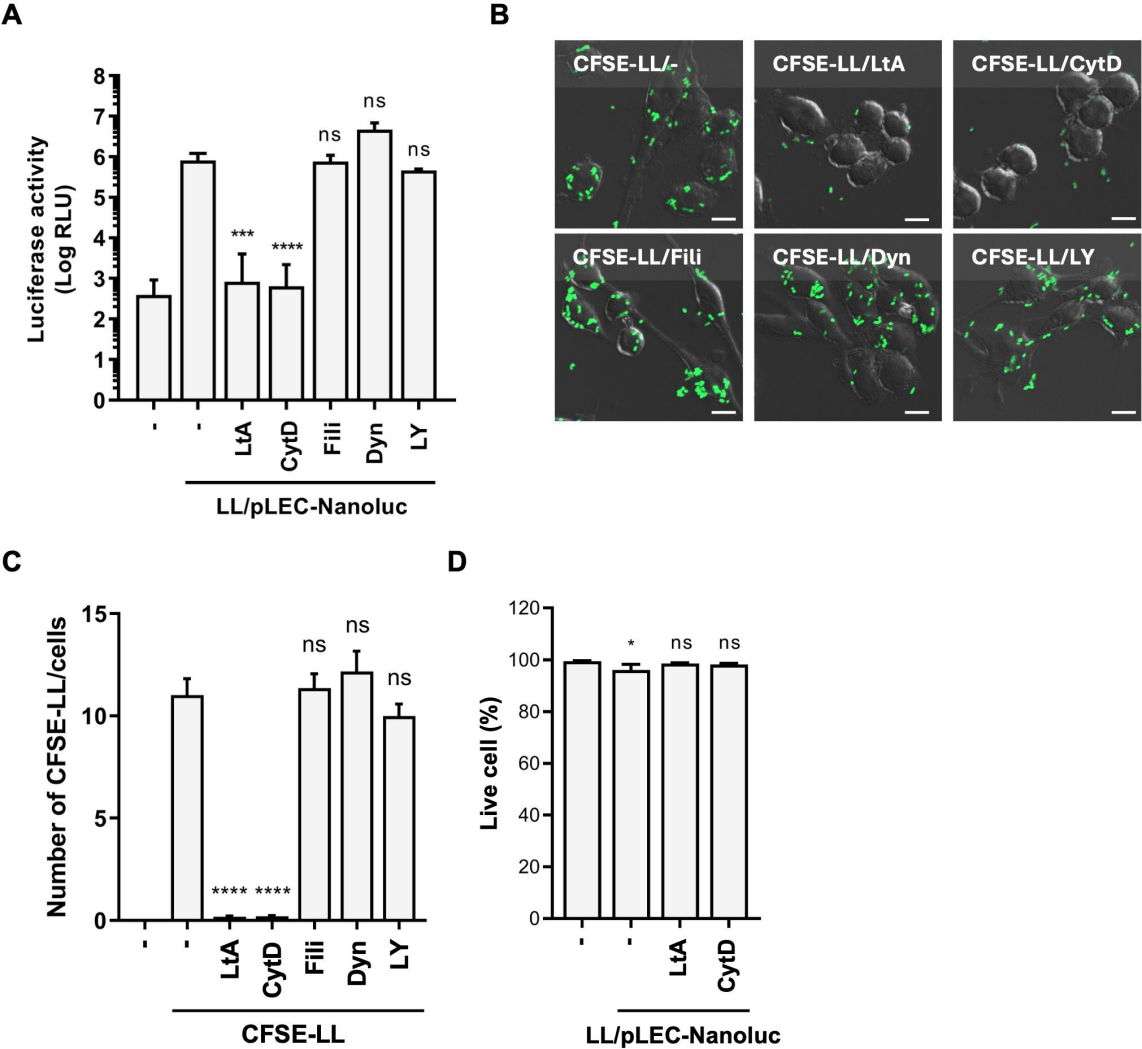
Effect of endocytosis inhibitors on the plasmid delivery. (**A**) Luciferase activity in RAW264.7 cells co-cultured with LL/pLEC-Nanoluc (cell-to-bacteria ratio 1:1,000) for 20 h in the presence of 2 µM LtA, 10 µM CytD, 2 µg/mL Filipin III (Fili), 10 µM Dynasore (Dyn), or 50 µM LY294002 (LY). Data are mean ± SEM (*n* = 3–4). (**B**) Fluorescent and phase-contrast images of RAW264.7 cells co-cultured with CFSE-labeled LL for 4 h with the inhibitors in panel **A**. Scale bars, 10 µm. (**C**) Quantification of cell-associated CFSE-LL (>200 cells/condition). (**D**) RAW264.7 cells co-cultured with LL/pLEC-Nanoluc for 26 h with 2 µM LtA or 10 µM CytD, stained with Hoechst 33342 and propidium iodide (PI). Percent live cells calculated from total (Hoechst^+^) and dead (PI^+^) counts (*n* = 4 wells/condition, 5 areas/well). Data are mean ± SEM. *P < 0.05, ***P < 0.001, ****P < 0.0001, ns, not significant, by one-way ANOVA with Dunnett’s test vs LL/pLEC-Nanoluc (**A, D**) or CFSE-LL (**C**) with no inhibitor.

To further investigate the internalization pathway of LL, we first assessed the endocytic capacities of these phagocytic and epithelial cell lines using the known markers FITC-CTB (general endocytosis [25, 26]), FD-2000 (macropinocytosis [27]), and F-Zymosan (phagocytosis [27]). After 2 h of incubation, confocal microscopy revealed that all cell lines internalized FITC-CTB and FD-2000 to varying degrees (Fig. S6), whereas F-Zymosan uptake was restricted to RAW264.7 and THP-1 cells. These results suggest that LL uptake by these phagocytic cell lines occurred predominantly via phagocytosis. To test this hypothesis, we examined the effects of various endocytic inhibitors on LL internalization by RAW264.7 cells. Actin polymerization inhibitors (Latrunculin A [LtA] and Cytochalasin D1 [CytD]), which block phagocytosis and macropinocytosis, completely inhibited luciferase expression (Fig. 4A) and LL internalization (Fig. 4B and C) without affecting cell viability (Fig. 4D). In contrast, the caveolar (Filipin III [28]), clathrin (Dynasore [29]), and macropinocytosis (LY294002 [30]) pathways were not affected. The specificity of these inhibitors was validated by measuring endocytic marker uptake (Fig. S7). These findings demonstrate that LL internalization and subsequent gene expression in RAW264.7 cells occur via phagocytosis.

### Post-phagocytic processes in plasmid delivery

**Fig 5:**
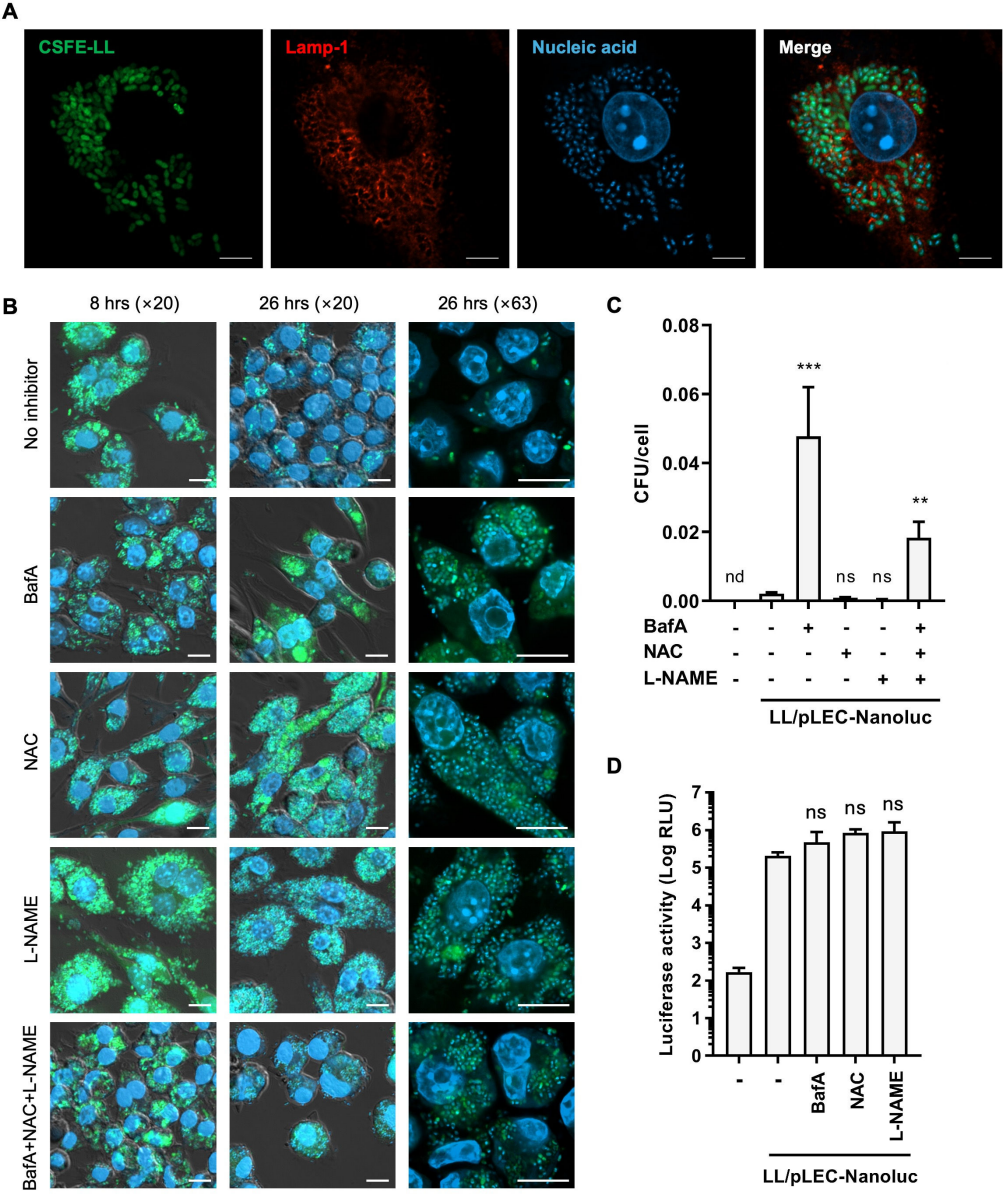
Effect of inhibitors of bactericidal mechanisms on degradation of phagocytosed *L. lactis* and plasmid transfer. (**A**) RAW264.7 cells co-cultured with CFSE-labeled LL (green, cell-to-bacteria ratio 1:1,000) for 8 h, stained with anti-Lamp-1 antibody (red) and Hoechst 33342 (blue). Images acquired with a ×63 objective. Scale bar, 5 µm. (**B**) RAW264.7 cells co-cultured with CFSE-labeled LL for 2 h, then treated with 40 nM Bafilomycin A1 (BafA), 2 mM N-acetylcysteine (NAC), 5 mM N-nitro-L-arginine methyl ester (L-NAME), or their combination for 8 or 26 h. Nuclei stained with Hoechst 33342. Images acquired with ×20 or ×63 objectives. Scale bar, 10 µm. (**C**) Viable LL (CFU per cell) in RAW264.7 cells after 26 h co-culture with LL/pLEC-Nanoluc. Cell lysates were plated on GM17 agar containing EM. Data are mean ± SEM (*n* = 4). nd, not detected. (**D**) Luciferase activity in RAW264.7 cells co-cultured with LL/pLEC-Nanoluc for 20 h with BafA, NAC, or L-NAME. Data are mean ± SEM (*n* = 4). **P < 0.01, ***P < 0.001, ns, not significant, by Kruskal-Wallis test (**C**) or one-way ANOVA with Dunnett’s test (**D**) vs LL/pLEC-Nanoluc with no inhibitor.

Previous studies using invasive bacterial vectors suggested that plasmid DNA is released following bacterial death after endosomal escape (31–33). However, the mechanisms underlying plasmid release by non-invasive vectors such as LL remain poorly understood (34). To address this, we examined the intracellular dynamics of LL in RAW264.7 cells. Confocal microscopy revealed that the number of CFSE-LL internalized by RAW264.7 cells increased over time, peaked at 8 h, and then slightly declined (Fig. S8A and B). After 26 h, the fluorescence signal from LL appeared to diminish, suggesting bacterial degradation (Fig. S8A). By 8 h, LL localized within Lamp-1^+^ phagosomes (Fig. 5A; Fig. S8C).

To determine the cellular mechanisms responsible for LL degradation, we treated RAW264.7 cells with inhibitors of lysosomal acidification (Bafilomycin A1 [BafA]), reactive oxygen species (ROS) (N-acetylcysteine [NAC]), and reactive nitrogen species (RNS) (N-nitro-L-arginine methyl ester [L-NAME]). The efficacy of these inhibitors was confirmed before experiments (Fig. S9A through C). Confocal microscopy showed that none of the inhibitors affected LL uptake at 8 h (Fig. 5B). However, at 26 h, cells treated with these inhibitors retained significantly more fluorescent LL than the untreated controls, indicating that lysosomal acidification, ROS, and RNS contributed to bacterial degradation (Fig. 5B, 26 h).

To assess bacterial viability, the cell lysates at 26 h were plated on GM17 agar with or without erythromycin (EM). Although the number of viable LL was low in NAC- or L-NAME-treated and untreated cells, significantly higher survival rates were observed in BafA-treated and cocktail-treated cells (Fig. 5C). Nonetheless, the number of viable bacteria was much lower than the fluorescent LL observed by microscopy, suggesting that most internalized LL were nonviable at this time point. Moreover, the number of colonies on EM-containing plates was comparable to those on EM-free plates in all conditions (Fig. S10A). This confirmed that virtually all viable LL retained the plasmid and that the lack of viability was not caused by plasmid loss. Despite their influence on LL degradation, none of the inhibitors significantly altered luciferase expression (Fig. 5D; Fig. S10B), suggesting that these bactericidal pathways were not essential for plasmid release or transfer.

## DISCUSSION

This study highlights the potential and current limitations of non-invasive LL as a mucosal DNA vaccine vector. We demonstrated that intranasal administration of non-invasive LL-carrying plasmid DNA can induce antigen-specific immune responses. In addition, *in vitro* assays showed that plasmid delivery from LL to host cells is dependent on phagocytic uptake rather than epithelial internalization.

Although the immunogenicity of LL-based DNA vaccines has been evaluated using genetically modified invasive strains (11–16), studies employing non-invasive LL strains for mucosal DNA vaccination remain limited, particularly in the context of intranasal administration. We found that intranasal delivery of non-invasive LL-vectored DNA vaccines elicited stronger antigen-specific immune responses than oral delivery when tested under identical conditions in terms of bacterial strain, plasmid, dose, number of administrations, and antibody assays. These findings suggest that the intranasal route may be more suitable for LL-based DNA vaccine delivery.

Cell-based assays revealed that LL was internalized and induced transgene expression in phagocytic cells, but not in epithelial cell lines. Inhibitor experiments confirmed that LL uptake occurred through actin-dependent phagocytosis. Confocal imaging showed that LL remained in the phagosomes over time, with bacterial degradation becoming evident after 26 h. Despite extensive uptake, only a small fraction of cells expressed the transgene, likely because of LL’s inability to escape from the phagosome, which is a known barrier to efficient gene transfer (35). Therefore, strategies to promote phagosomal escape, such as the expression of endosomolytic peptides (36, 37), may improve delivery efficiency. However, it should be noted that our study employed transformed macrophage-like cell lines (RAW264.7 and THP-1) as models. Although these are useful for mechanistic studies, they may not fully reflect the behavior of primary tissue-resident immune cells *in vivo*. Nonetheless, our additional experiments using primary splenocytes support that LL-mediated plasmid delivery can also occur in physiologically relevant phagocytic cells. Future studies, including *in situ* analysis or depletion of phagocytic cells with chemicals such as clodronate liposomes (38, 39), will be important for validating the observed mechanisms.

*In vivo*, most of the nasally administered LL remains on the epithelial surface, with only a small fraction reaching the underlying tissues where phagocytic cells reside. We assume this finding reflects a rare translocation through incidental, phagocytosis-independent routes across the epithelial barrier, such as transient intercellular gaps associated with local inflammation (40) or transcytosis via epithelial cells (41, 42), as reported for certain bacterial species in the gut mucosa. In addition, the colocalization of CFSE-LL with Ly6G^+^ regions lacking nuclei in nasal tissues suggests the presence of NET-like structures (43, 44), which may represent neutrophil extracellular traps capturing bacteria at mucosal surfaces. Such entrapment likely limits the accessibility of LL to epithelial or subepithelial cells. This spatial limitation, combined with the low efficiency of plasmid delivery, likely restricts the overall immunogenicity of the LL-vectored vaccines. Enhancing tissue penetration or more effectively targeting phagocytes could further improve vaccine performance.

We attempted to identify phagocytic cell subsets interacting with LL by immunofluorescence staining, but marker detection was technically limited by the decalcification process required for nasal tissue preparation. Future work using non-decalcifying imaging or flow cytometric analysis of nasal cell suspensions will help overcome this limitation. To identify recipient cells, we also attempted to detect EGFP-expressing cells in nasal tissues; however, no EGFP-positive cells were observed. This negative result likely reflects limited transgene expression at the tissue level, spatially restricted expression that is diluted in tissue sections, and the lower sensitivity of direct EGFP fluorescence detection *in situ*. In addition, without a tissue-positive control for EGFP detection, we cannot exclude a detection limit for our immunofluorescence protocol.

In conclusion, this study provides a proof of concept that non-invasive *Lactococcus lactis* can be used for intranasal DNA vaccine delivery and that phagocytosis represents a critical step in gene transfer. While the overall transgene expression and immunogenicity remain modest, these findings establish a mechanistic basis for improving LL-based mucosal vaccines. Further optimization, such as engineering LL for improved intracellular release, will provide a foundation for the future development of practical and effective mucosal DNA vaccines.

## MATERIALS AND METHODS

### Bacterial strains and plasmid

*E. coli* strain BL21 was cultured in LB medium at 37°C with shaking. LL NZ9000 (MoBiTec) was cultured in M17 medium supplemented with 0.5% glucose at 30°C without shaking. EM was added at 200 µg/mL for *E. coli* and 5 µg/mL for LL if necessary. The LL-*E. coli* shuttle plasmid, pLEC, was previously constructed (21) and contains a pAMβ1 replicon ensuring plasmid maintenance in LL (22), an EM resistance gene, a CMV promoter, and a chimeric intron from human β-globin and immunoglobulin heavy chain genes at the 5′-untranslated region for efficient eukaryotic gene expression (Fig. S1A). The EGFP and Nanoluc genes were cloned into pLEC, as previously described (21). These plasmids were introduced into electrocompetent LL cells to generate the LL/pLEC-EGFP and LL/pLEC-Nanoluc strains. To produce the recombinant EGFP protein, the EGFP gene was cloned into the pGEX plasmid to construct the GST-tagged EGFP expression plasmid pGEX-EGFP. *E. coli* BL21 cells were transformed with pGEX-EGFP, and EGFP expression was induced with 1 mM isopropyl β-D-thiogalactopyranoside (Fujifilm Wako) at 37°C for 3 h. Bacterial cells were collected by centrifugation and lysed by sonication. GST-EGFP was purified using Glutathione Sepharose 4B (Merck), and the protein concentration was determined by densitometry of Coomassie-stained SDS-PAGE gels using ImageLab software (Bio-Rad). Purified proteins were stored at −80°C until use.

### Immunization

Log-phase cultures of NZ9000 or LL/pLEC-EGFP were harvested, washed, and resuspended in phosphate-buffered saline (PBS) at concentrations of 1 × 10^10^ CFU/mL (oral administration, *n* = 7 for PBS, *n* = 10 for LL group) and 1 × 10^11^ CFU/mL (nasal administration, *n* = 4 for PBS, *n* = 7 for NZ9000, *n* = 9 for LL group). C57BL/6 mice (6-week-old, female, SLC Japan) received 2 × 10⁹ CFU of bacteria via oral (200 µL) or intranasal (20 µL; 10 µL per nostril) routes. Oral immunization was performed without anesthesia, whereas intranasal administration was performed under isoflurane inhalation. Both groups were immunized on 3 consecutive days every 2 weeks (days 0–2, 14–16, 28–30, and 42–44). For comparison, a group of mice received intramuscular injections of 10 µg purified pLEC-EGFP plasmid (prepared using an endotoxin-free plasmid purification kit, Promega) in 50 µL PBS (*n* = 5). Injections were performed on days 1, 11, 21, and 31 under anesthesia induced by intraperitoneal injection of medetomidine (0.3 mg/kg), midazolam (4 mg/kg), and butorphanol (5 mg/kg). Owing to the anticipated greater variability in the LL group, the larger sample sizes were assigned. In contrast, the PBS and intramuscular groups are well-established control models with relatively low variability in our pilot study, and therefore, small group sizes were used in accordance with the principle of reduction in animal use. On day 51, blood, feces, and nasal wash samples were collected to measure EGFP-specific antibody titers using ELISA. Sera were prepared from the blood specimens by centrifugation (800 × *g*, 20 min, 4°C) after clotting. Fecal pellets (100 mg/mL in PBS with complete protease inhibitor cocktail; Roche Diagnostics) were homogenized and centrifuged at 20,000 × *g* for 10 min at 4°C to obtain fecal extracts. Nasal washes were collected by flushing the nasal cavity with 400 µL PBS, followed by centrifugation at 800 × *g* for 20 min at 4°C. All samples were stored at −20°C until further analysis.

### ELISA

To measure EGFP-specific antibody titers, 96-well plates (MaxiSorp, Nunc) were coated with 100 ng/well of purified GST-tagged EGFP. Sera, fecal extracts, and nasal washes, diluted with PBS containing 0.1% Tween-20 and 5% skim milk (1:100, 1:2, and 1:2, respectively), were then added and incubated for 1 h at 37°C. Horseradish peroxidase-conjugated polyclonal goat anti-mouse IgG or IgA antibodies (Southern Biotech) were added and further incubated for 1 h at 37°C. Plates were developed using an o-phenylenediamine substrate; the reaction was stopped by adding H_2_SO_4_, and the OD_492-650_ values were measured.

### Protein extraction and SDS-PAGE

Log-phase cultures of LL/pLEC-EGFP and LL/pLEC-Nanoluc were harvested and washed twice with ice-cold PBS. Total cell lysates were prepared by resuspending the pellets in SDS buffer, followed by sonication. For Western blotting, equal amounts of lysates (equivalent to 2 × 10^8^ CFU) were separated by SDS-PAGE and transferred onto a PVDF membrane. The membrane was blocked with 5% skim milk in Tris-buffered saline containing 0.1% Tween-20 (TBST) for 1 h at room temperature, followed by incubation with anti-EGFP polyclonal antibody (purchased from MBL, 1:1,000 dilution) overnight at 4°C. After washing with TBST, the membranes were incubated with horseradish peroxidase-conjugated secondary antibodies (1:2,000 dilution) for 1 h at room temperature. Signals were visualized using chemiluminescence reagents (Immunostar LD, Fujifilm Wako) and captured with a Chemidoc Touch (Bio-Rad). For Coomassie Brilliant Blue (CBB) staining, SDS-PAGE gels run in parallel with those used for Western blotting were stained with CBB R-250 solution.

### Localization of intranasally administered LL

A log-phase culture of LL/pLEC-EGFP was harvested, stained with 50 µM CFSE (DOJINDO) at 37°C for 30 min in the dark with shaking, and washed with PBS to remove the excess dye. C57BL/6 mice (6-week-old, female, SLC Japan) were anesthetized with isoflurane and intranasally administered 2 × 10^9^ CFU of CFSE-labeled LL/pLEC-EGFP. Mice were euthanized 4 h post-administration by cervical dislocation in accordance with institutional guidelines, and their heads were collected and fixed overnight in 4% paraformaldehyde (PFA) at 4°C. The fixed heads were decalcified (Super Decalcifier I, Polysciences Inc.) for 6 h, embedded in OCT compound (Sakura Finetek, Japan), flash-frozen, and sectioned at 10 µm thickness. Sections were stained with anti-Ly6G antibody (Thermo Fisher Scientific) and Alexa Fluor 594-conjugated secondary antibody (Thermo Fisher Scientific), counterstained with Hoechst 33342, and treated with autofluorescence quenching reagent (Vector TrueVIEW Kit, Vector Laboratories). The slides were mounted with VECTASHIELD Vibrance Antifade Mounting Medium (Vector Laboratories) and imaged using a BZ-X810 fluorescence microscope (Keyence) or an LSM900 confocal microscope (ZEISS).

### Detection of EGFP-expressing cells in nasal tissues

C57BL/6 mice (6-week-old, female, SLC Japan) were anesthetized with isoflurane and intranasally administered PBS or 2 × 10^9^ CFU of LL/pLEC-EGFP. The administration was performed for 3 consecutive days. Three days after the final dose, the mice were euthanized by cervical dislocation, as previously described. Nasal tissue sections were prepared, stained with an anti-EGFP polyclonal antibody and Alexa Fluor 488-conjugated secondary antibody (Thermo Fisher Scientific), counterstained with Hoechst 33342, and analyzed.

### Reagents

LtA, CytD, NAC, and L-NAME were obtained from Wako; Filipin III and Dynasore from Cayman Chemicals; BafA from Merck; and LY294002 from Cell Signaling Technology. LtA, CytD, Filipin III, Dynasore, LY294002, and BafA were dissolved in DMSO at 1 mM, 10 mM, 1 mM, 10 mM, 25 mM, and 200 µM, respectively, and stored at −20°C. Immediately before use, NAC and L-NAME were dissolved in the cell culture medium to final concentrations of 2 and 5 mM, respectively. The inhibitors were added to the culture medium 1 h before adding LL. Fluorescent endocytosis tracers FITC-conjugated dextran 2,000 kD (FD-2000, TdB Labs), FITC-conjugated Cholera toxin B subunit (FITC-CTB, Sigma-Aldrich), and Acidi-Fluor Zymosan A (F-Zymosan, GORYO CHEMICAL) were used. The final concentrations of the inhibitors are indicated in the figure legends.

### Cells

The murine macrophage-like cell line RAW264.7 (Riken Cell Bank) and human monocytic cell line THP-1 (Riken Cell Bank) were cultured in RPMI 1640 supplemented with 10% fetal bovine serum (FBS) at 37°C in 5% CO_2_. The murine colonic epithelial cell line CMT-93 (ATCC) was cultured in low-glucose Dulbecco’s modified Eagle’s medium (DMEM) supplemented with 10% FBS at 37°C in 5% CO_2_. The human retinal pigment epithelial cell line ARPE-19 (ATCC) was cultured in high-glucose DMEM/Ham’s F-12 supplemented with 10% FBS at 37°C in 5% CO_2_.

### Luciferase assay to measure phagocytic activity

A log-phase culture of LL/pLEC-Nanoluc cells was washed with PBS and resuspended in cell culture medium. RAW264.7, THP-1, CMT-93, and ARPE-19 cells were seeded into 96-well plates at a density of 2 × 10^4^ cells per well. Bacterial suspensions were added at cell-to-bacterial ratios of 1:200, 1:1,000, or 1:5,000. After 2 h, the medium was replaced with fresh medium containing 20 µg/mL gentamicin (GM), and the cells were incubated for the indicated durations. Cells were lysed with passive lysis buffer (Promega), and luciferase activity was measured using the Nano-Glo Luciferase Assay System (Promega) and a GloMax-Multi Detection Plate Reader (Promega).

### Co-culture of LL/pLEC-Nanoluc with mouse splenocytes

Fresh splenocytes were isolated from C57BL/6 mice using mechanical dissociation through a 70 µm cell strainer, followed by red blood cell lysis. Cells were suspended in RPMI 1640 supplemented with 10% FBS and plated in 12-well plates at 4 × 10⁶ cells per well with 4 × 10^7^ CFU of LL/pLEC-Nanoluc. After 2 h of incubation at 37°C, non-adherent cells and bacteria were removed, and the adherent splenocytes were then incubated overnight in medium containing 20 µg/mL GM. After washing out residual non-adherent cells, adhered cells were lysed with passive lysis buffer (Promega). Luciferase activity in cell lysates was measured as described above.

### Flow cytometry

A log-phase culture of LL/pLEC-EGFP was washed with PBS and resuspended in cell culture medium. RAW264.7 cells were seeded in a 12-well plate at a density of 2 × 10^5^ cells per well. RAW264.7 cells were co-cultured with LL/pLEC-EGFP as described above. After incubation for 20 h, cells were washed with PBS and harvested using a cell scraper. The dead cells were stained with Fixable Viability Dye eFluor 780 (Thermo Fisher Scientific). EGFP^+^ cells were analyzed using flow cytometry (Cytoflex-S, Beckman Coulter).

### Confocal microscopy

Cells were seeded in an eight-well chamber slide at 4.5 × 10^4^ cells per well. CFSE-labeled bacteria, prepared as described above, were added at a cell-to-bacteria ratio of 1:1,000 and incubated for 2 h, followed by additional incubation with GM for the indicated durations. The cells were washed with PBS, fixed with 4% PFA for 15 min at room temperature, stained with anti-Lamp-1 (clone 1D4B; Santa Cruz Biotechnology) and anti-rat IgG-Alexa 594 (Thermo Fisher Scientific), or fixed with methanol for 5 min at −20°C, stained with anti-EGFP polyclonal antibody (MBL), anti-rabbit IgG-Alexa 594 (Thermo Fisher Scientific), and Hoechst 33342, and mounted with ProLong Gold antifade reagent (Thermo Fisher Scientific). Images were obtained using an LSM900 confocal microscope (ZEISS). The fluorescence intensity per cell, bacterium, and background was measured using the ZEN software (ZEISS). The number of cell-associated CFSE-LLs was calculated by subtracting the background fluorescence intensity per cell area from the fluorescence intensity per cell and dividing it by the background-corrected fluorescence intensity per bacterium based on measurements from at least 200 cells across four distinct fields/conditions.

### Cell viability assay

RAW264.7 cells were seeded in a 96-well plate at a density of 2 × 10^4^ cells per well. The bacterial suspension was added at a cell-to-bacteria ratio of 1:1,000 and incubated for 2 h, followed by an additional incubation with GM for 18 h. The cells were then washed with PBS and stained with Hoechst 33342 and propidium iodide (PI). Fluorescent images were captured using a ZOE Fluorescent Cell Imager (Bio-Rad). The number of viable (Hoechst 33342^+^) and dead (PI^+^) cells in at least five fields per well was automatically counted using ImageJ software (National Institutes of Health, USA).

### ROS assay

After co-culturing RAW264.7 cells and LL for 24 h in the presence or absence of 2 mM NAC in a six-well plate, total ROS levels were measured using the ROS Assay Kit—Photo-oxidation Resistant DCFH-DA (DOJINDO), according to the manufacturer’s instructions. Briefly, the cells were harvested and incubated with DCFH-DA for 30 min at 37°C. The cells were washed with Hank’s balanced salt solution to remove excess probes, and fluorescence was measured using Cytoflex-S.

### NO assay

After co-culturing RAW264.7 cells and LL for 24 h in the presence or absence of 5 mM L-NAME in a 96-well plate, the concentration of nitrite, a final metabolite of RNS, was measured in the culture supernatants using the Griess method, as described previously (45).

### Phagosome acidification

RAW264.7 cells were seeded in a 96-well plate at a density of 2 × 10^4^ cells per well. After 1 h of treatment with 40 nM BafA, 20 µg/mL F-Zymosan was added to the cells. Fluorescent images were captured using a ZOE Cell Imager before and at 1, 3, and 5 h after probe addition. The fluorescence intensity of the images was measured using ImageJ software.

### Determination of the number of live LL in RAW264.7 cells

RAW264.7 cells were seeded in a 24-well plate at a density of 8 × 10^4^ cells per well. Cells were treated with 2 mM NAC, 5 mM L-NAME, 40 nM BafA, or a cocktail of these three inhibitors for 1 h, followed by the addition of LL. After co-culturing for 2 h, the culture media were exchanged and incubated for an additional 24 h with 20 µg/mL GM and inhibitors. Cells were washed with PBS and harvested using a cell scraper. Cells were counted using a hemocytometer and lysed in PBS containing 0.1% Triton X-100. Lysates were plated on GM17 plates with or without EM and incubated for 48 h, followed by counting of the number of colonies.

### Statistical analysis

Data were analyzed using Student’s *t*-test, Mann-Whitney U-test, Kruskal-Wallis test, or one-way ANOVA followed by Tukey’s or Dunnett’s test using GraphPad Prism 8 (GraphPad Software). Statistical significance was set at *P* < 0.05. Details of the statistical methods applied to each data set are provided in the figure legends.

## SUPPLEMENTAL MATERIAL

Supplemental material iai.00460-25-s0001.pdf Fig. S1 to S10.

ASM does not own the copyrights to Supplemental Material that may be linked to, or accessed through, an article. The authors have granted ASM a non-exclusive, world-wide license to publish the Supplemental Material files. Please contact the corresponding author directly for reuse.
