# Supplementary material for: Immunogenicity and plasmid delivery pathways of non-invasive Lactococcus lactis-vectored mucosal DNA vaccination
Source: Infect Immun. 2025 Nov 28;94(1):e00460-25. doi: 10.1128/iai.00460-25 (PMC12798020; doi:10.1128/iai.00460-25)
Supplement: Supplemental material — Fig. S1 to S10. [file iai.00460-25-s0001.pdf]

Fig. S1

A

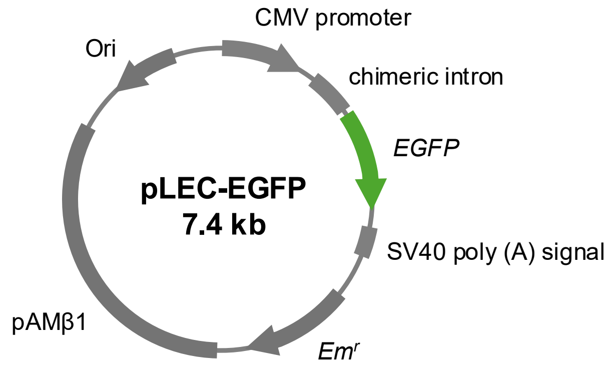

B

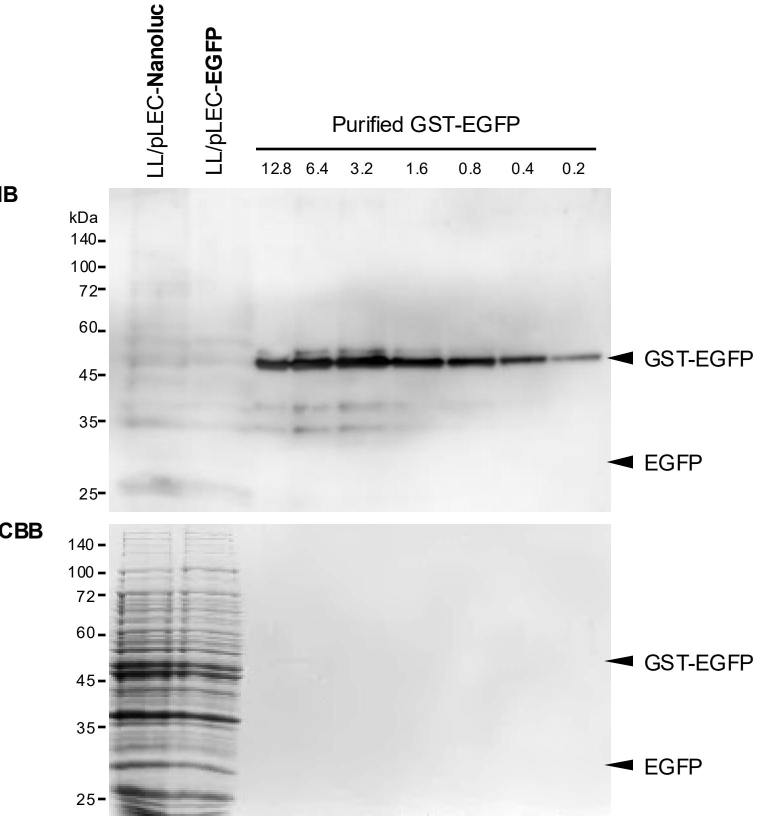

Fig. S1. Characterization of the pLEC-EGFP plasmid.
(A) Plasmid map of pLEC-EGFP. The construct contains a CMV promoter and chimeric intron upstream of the EGFP coding sequence, followed by an SV40 poly(A) signal, designed for efficient expression in mammalian cells. Em^{r}, erythromycin resistance marker; pAMβ1, replicon ensuring plasmid maintenance in *L. lactis*; Ori, replication origin for *E. coli*. **(B)** Immunoblotting with anti-EGFP antibody (top) and CBB staining (bottom) of bacterial lysates equivalent to 1 × 10^{8} CFU of LL/pLEC-EGFP or LL/pLEC-Nanoluc, and purified GST-EGFP (ng/lane). Predicted band sizes of GST-EGFP (52 kDa) and EGFP (28 kDa) are shown. No detectable EGFP expression was observed in LL/pLEC-EGFP, consistent with the plasmid being designed for mammalian expression.

Fig. S2

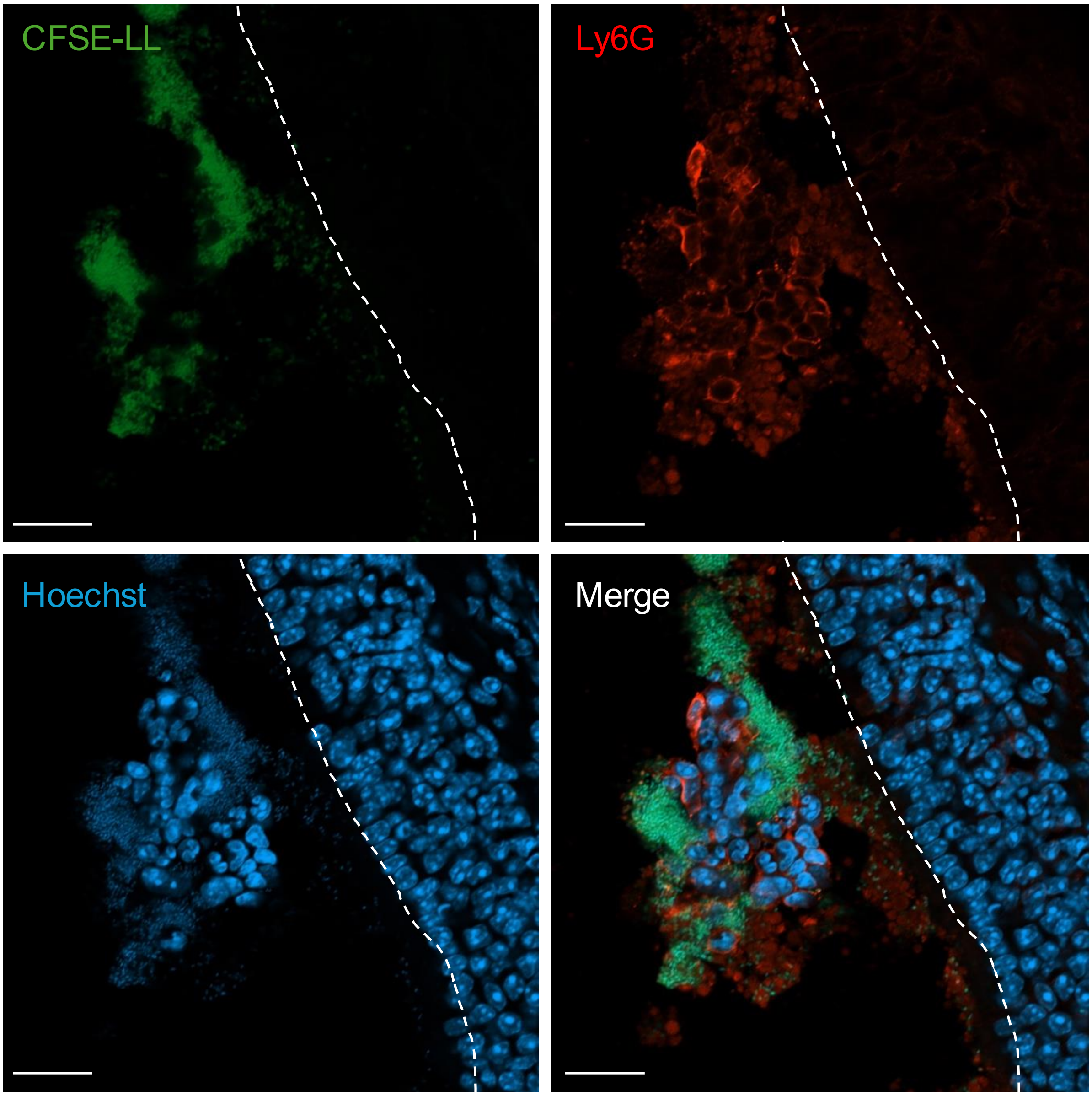

Fig. S2. CFSE-labeled LL signals colocalized with Ly6G⁺ regions.
Representative confocal fluorescence images showing the colocalization of CFSE-labeled LL (green) with Ly6G⁺ cells (red) in nasal tissues 4 h after intranasal administration. Nuclei were counterstained with Hoechst 33342 (blue). Most CFSE-LL signals were localized within Ly6G⁺ regions lacking intact nuclei, suggestive of trapping within NET-like structures formed in the nasal lumen. Dashed lines indicate interface between epithelia and lumen. Scale bars, 20 μm.

Fig. S3

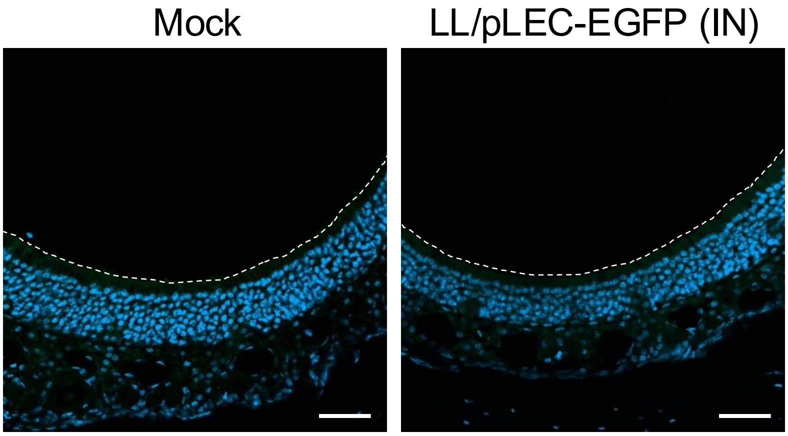

Fig. S3 EGFP-expression was not detected in the nasal tissues of mice administered with LL/pLEC-EGFP.
Mice were intranasally administered PBS (Mock) or 2 × 10^9 CFU of LL/pLEC-EGFP for immunofluorescent analysis. Nasal tissue sections stained with an anti-EGFP antibody (green, not detected) and Hoechst 33342 (blue). Dashed lines indicate interface between epithelia and lumen. Scale bars indicate 50 µm.

Fig. S4

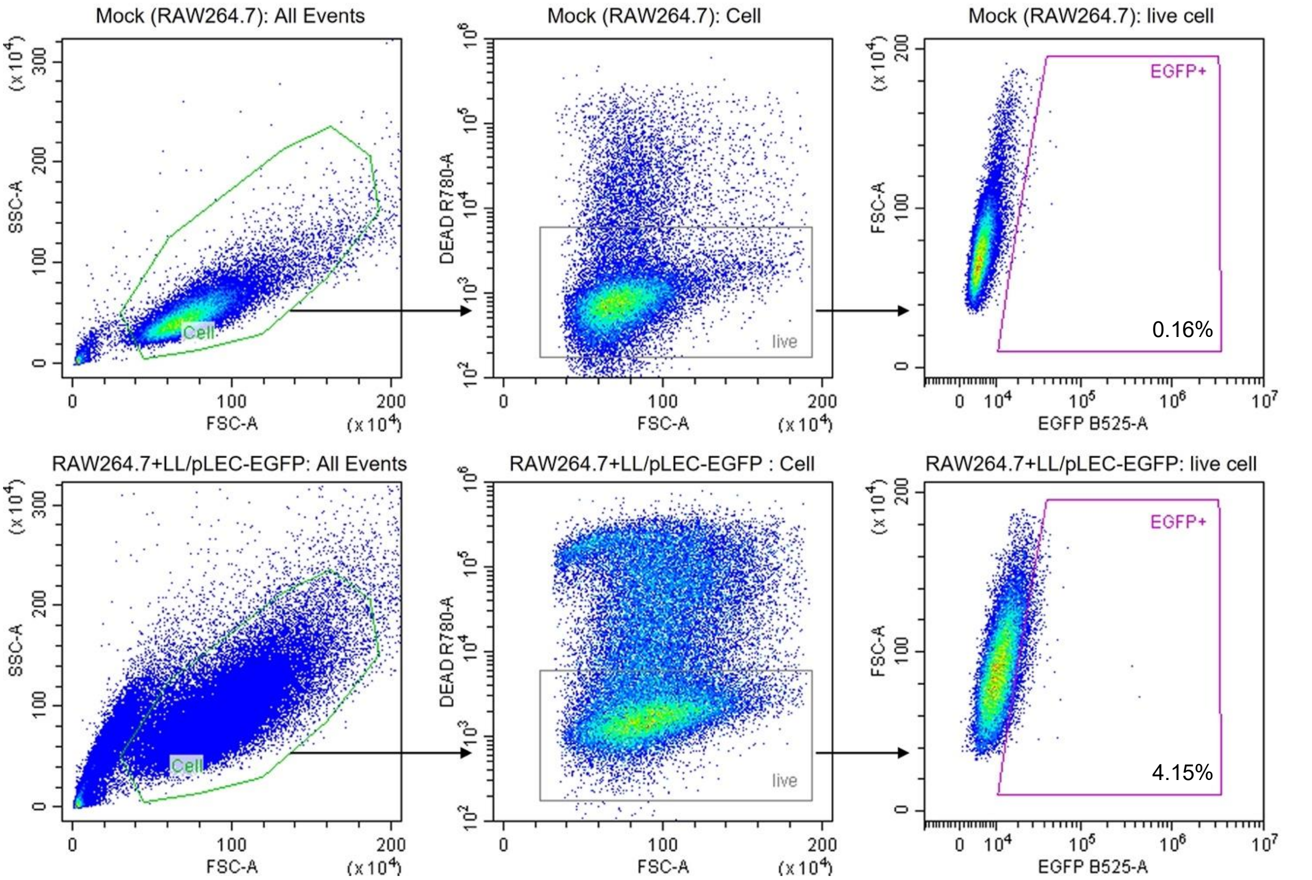

Fig. S4. Gating strategy used for flow cytometric analysis of EGFP⁺ RAW264.7 cells co-cultured with LL/pLEC-EGFP.

Gating strategy used to quantify EGFP⁺ RAW264.7 cells. Cells were first gated on FSC-A/SSC-A to exclude debris, followed by gating on FVD780⁻ viable cells. EGFP⁺ cells were then identified based on EGFP fluorescence versus FSC-A. The same gating strategy was applied to both mock (RAW264.7 cells without co-culture) and LL/pLEC-EGFP co-cultured samples. RAW264.7 cells exposed to LL/pLEC-EGFP showed increased FSC and SSC values, consistent with cellular activation and morphological enlargement upon bacterial exposure. The higher proportion of dead cells in the LL/pLEC-EGFP group likely reflects strong adherence of activated macrophages requiring more vigorous scraping during collection, rather than true cytotoxicity. This interpretation is supported by Hoechst/PI staining (Fig. 4D), which confirmed that co-culture with LL did not induce cell death.

Fig. S5

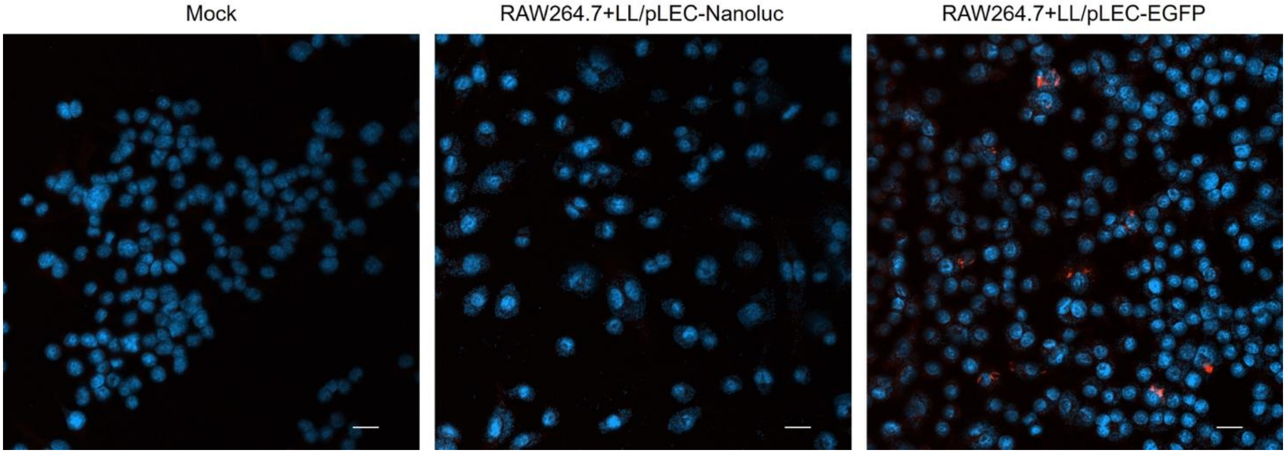

Fig. S5. Immunofluorescence detection of EGFP expression in RAW264.7 cells co-cultured with LL/pLEC-EGFP.

RAW264.7 cells were co-cultured for 20 h with LL/pLEC-EGFP or LL/pLEC-Nanoluc (negative control). After methanol fixation, cells were stained with a rabbit polyclonal anti-EGFP antibody followed by Alexa Fluor 594–conjugated anti-rabbit IgG (red). Nuclei were counterstained with Hoechst 33342 (blue). Immunofluorescence staining revealed EGFP expression in a subset of cells co-cultured with LL/pLEC-EGFP, whereas no signal was observed in the Mock (RAW264.7 cells without co-culture) and LL/pLEC-Nanoluc control. Scale bars, 10 μm.

Fig. S6

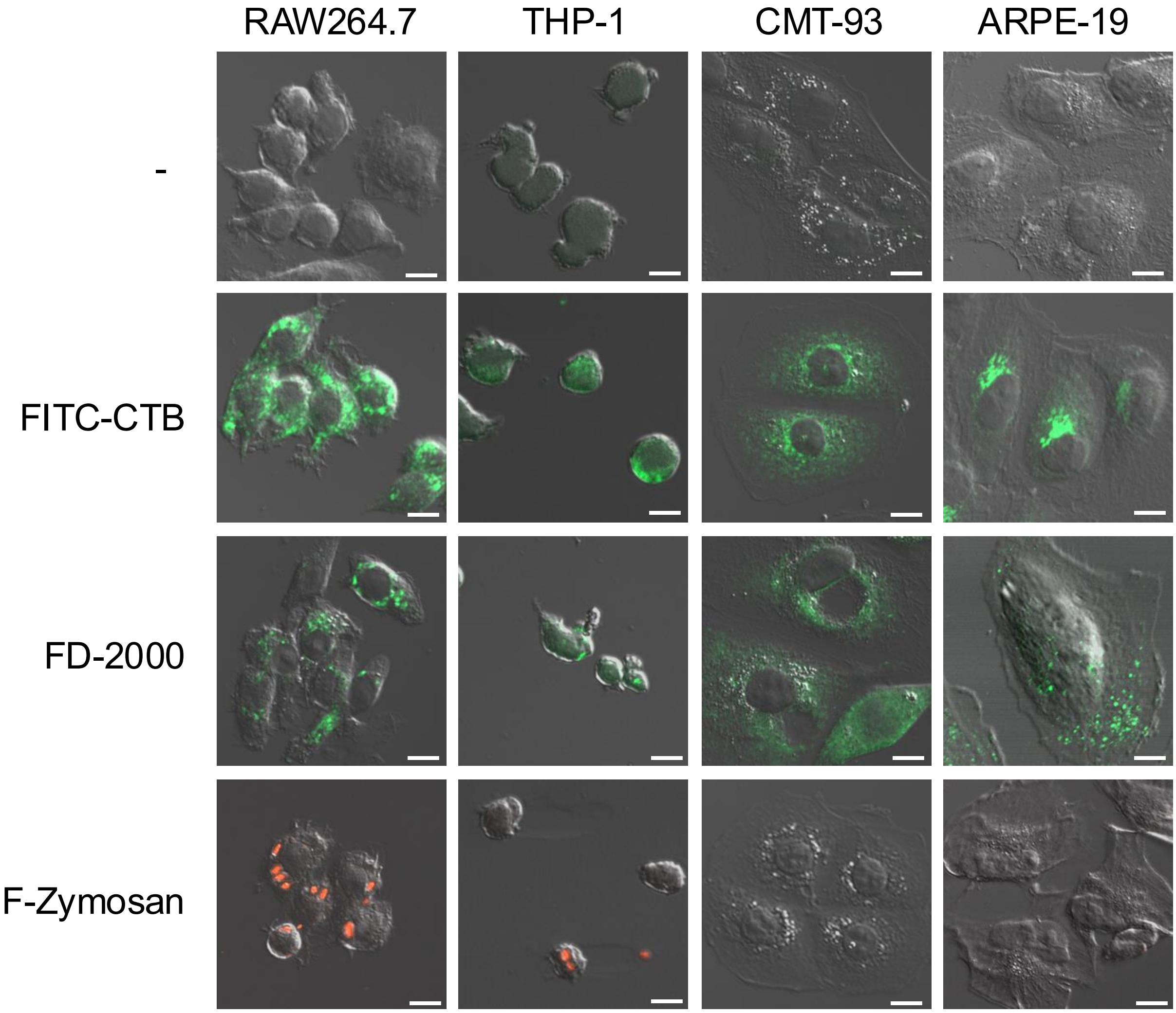

Fig. S6 Internalization of fluorescent endocytosis tracers by different cells.
RAW264.7, THP-1, CMT-93, and ARPE-19 cells were cultured in chamber slides. FITC-conjugated Cholera toxin B subunit (FITC-CTB, 5 µg/mL), FITC-conjugated dextran 2,000 kD (FD-2000, 0.5 µg/mL), or Acidi-Fluor-Zymosan A (F-Zymosan, 20 µg/mL) were added to the cells and incubated for 2 h. Fluorescent and phase-contrast images were obtained with using a confocal microscopy. Bars indicate 10 µm.

Fig. S7

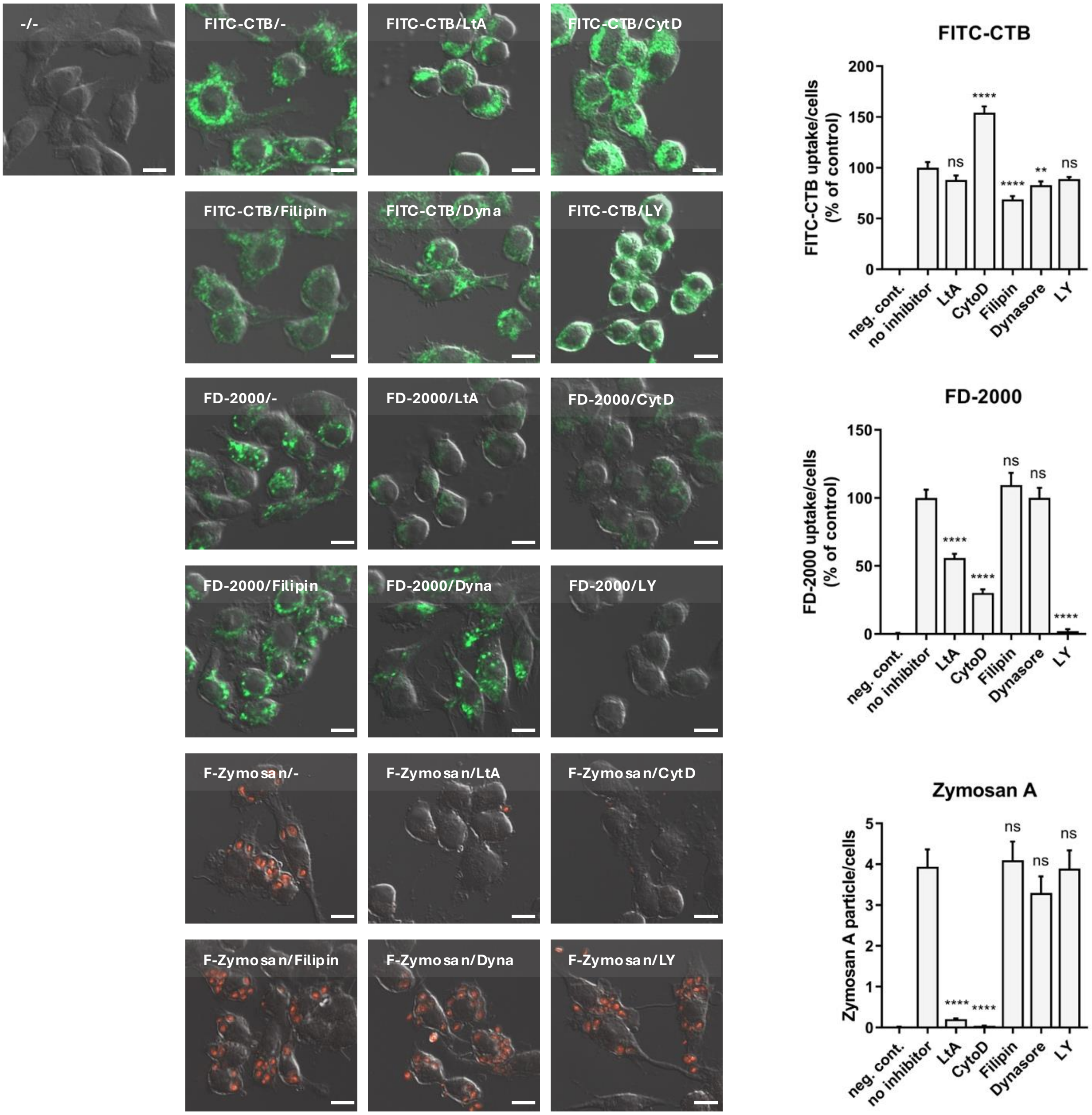

Fig. S7 Internalization of fluorescent endocytosis tracers by different cells.
RAW264.7 cells were cultured on chamber slides. Latrunculin A (LtA, 2 μM), Cytochalasin D (CytD, 10 μM), Filipin III (Fili, 2 μg/mL), Dynasore (Dyn, 10 μM), or LY294002 (LY, 50 μM) were added to the cells and incubated for 30 min. FITC-conjugated Cholera toxin B subunit (FITC-CTB, 5 μg/mL), FITC-conjugated dextran 2,000 kD (FD-2000, 0.5 μg/mL), or Acidi-Fluor-Zymosan A (F-Zymosan A, 20 μg/mL) were added to the cells and incubated for 2 h. Fluorescent and phase-contrast images were obtained using confocal microscopy. Bars indicate 10 μm. The fluorescence intensity of FITC-CTB and FD-2000/cell and the number of Zymosan A particles/cell were measured. Data are expressed as mean ± SEM. **p<0.01, ****p<0.0001, ns; not significant, by one-way ANOVA followed by Dunnett's test (vs. fluorescent tracer with no inhibitor).

Fig. S8

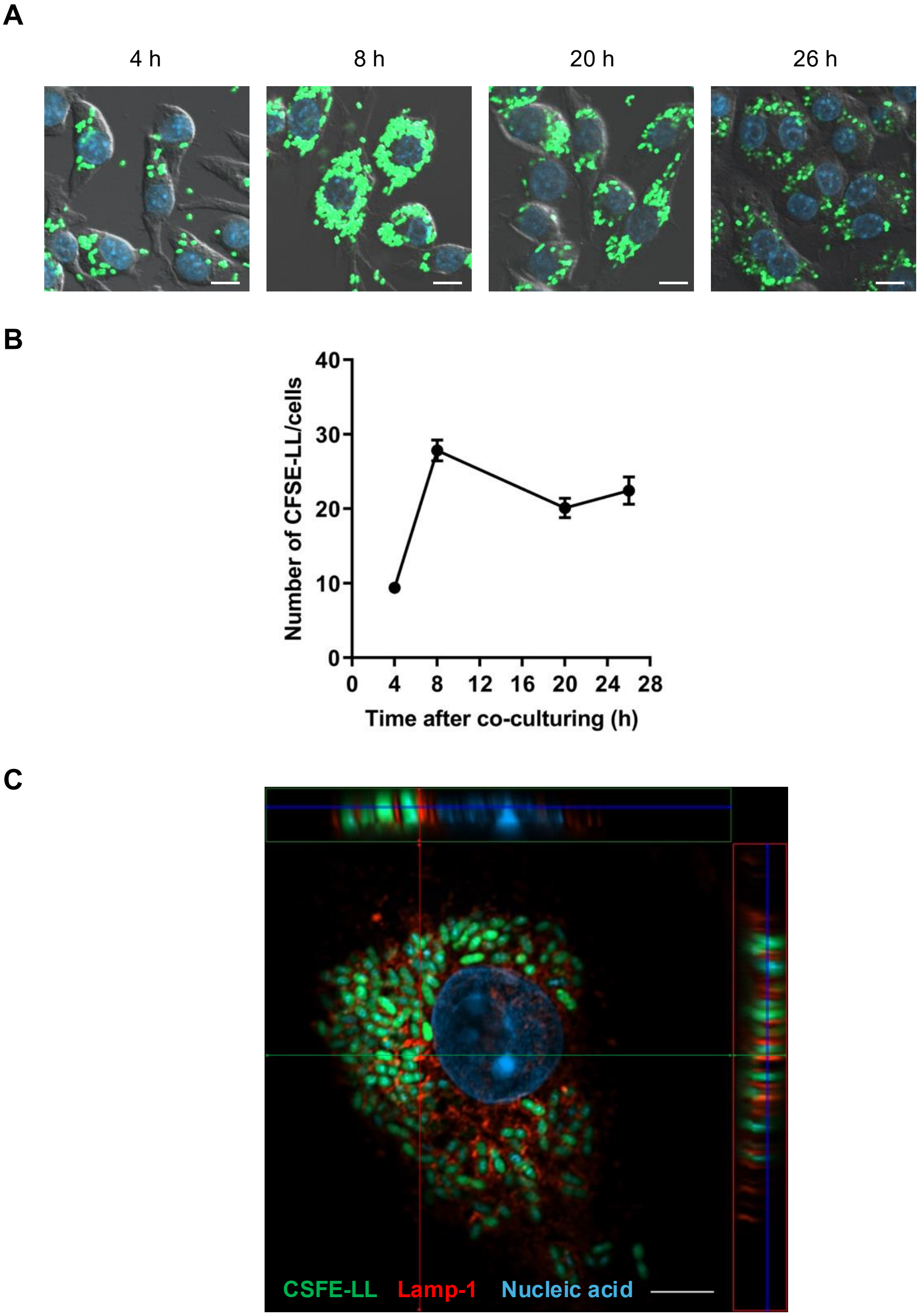

Fig. S8 Internalization of CFSE-LL by RAW264.7 cells

(A) Fluorescent and phase contrast images of RAW264.7 cells co-cultured with CFSE-LL (cell-to-bacteria ratio was 1,000) for the indicated durations. Nuclei were stained with Hoechst 33342 (blue). Scale bars indicate 10 μm. **(B)** For each time point, the number of cell-associated CFSE-LL in more than 200 cells were enumerated (mean ± SEM). **(C)** Ortho image of RAW264.7 cells co-cultured with CFSE-labeled LL (green) (cell-to-bacteria ratio was 1:1,000) for 8 h. Cells were stained with anti-Lamp-1 antibody (red) and Hoechst 33342 (blue). The fluorescent image was obtained with a ×63 objective lens by Z-stack mode (0.3 μm × 14 slices). Scale bar indicates 5 μm.

Fig. S9

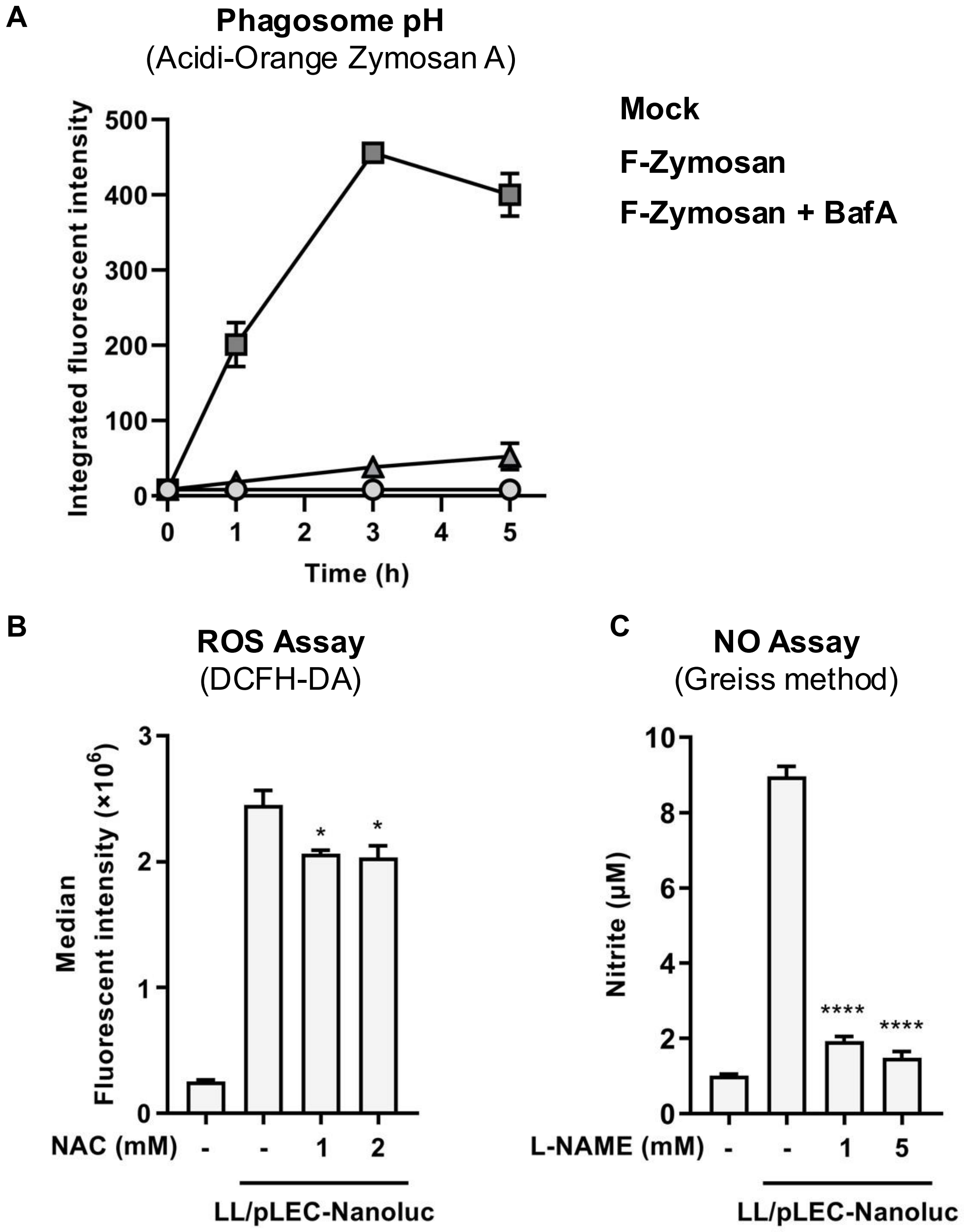

Fig. S9 Validation of the effects of inhibitors for bactericidal mechanisms

(A) RAW264.7 cells were cultured with Acidi-Fluor-Zymosan A (F-Zymosan, 20 μg/mL). Bafilomycin A1 (BafA, 40 nM) was added to the medium 1 h before addition of F-Zymosan. Red fluorescent images were obtained before and 1, 3, and 5 h after the addition of F-Zymosan. Integrated fluorescent intensity was calculated. Data are expressed as mean ± SEM (n=3). **(B)** RAW264.7 cells were cultured with LL/pLEC-Nanoluc (cell to bacteria ratio was 1:1,000) for 2 h with 1 or 2 mM NAC, followed by 18 additional h with gentamicin and NAC. Cells were washed, harvested, and incubated with DCFH-DA. Fluorescent intensity of oxidized DCFH-DA was measured by flow cytometry. Data are expressed as mean ± SEM (n=3). *$p<0.05$, by one-way ANOVA followed by Dunnett's test (vs. LL/pLEC-Nanoluc with no inhibitor). **(C)** RAW264.7 cells were cultured with LL/pLEC-Nanoluc (cell to bacteria ratio was 1:1,000) for 2 h with 1 or 5 mM L-NAME, followed by 18 additional h with gentamicin and L-NAME. Concentrations of nitrite in the culture supernatants were measured using the Greiss method. Data are expressed as mean ± SEM (n=3). ****$p<0.0001$, by one-way ANOVA followed by Dunnett's test (vs. LL/pLEC-Nanoluc with no inhibitor).

Fig. S10

A

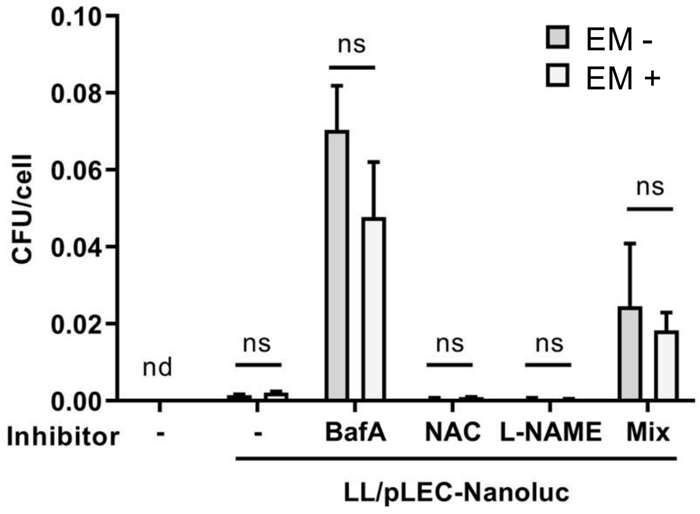

B

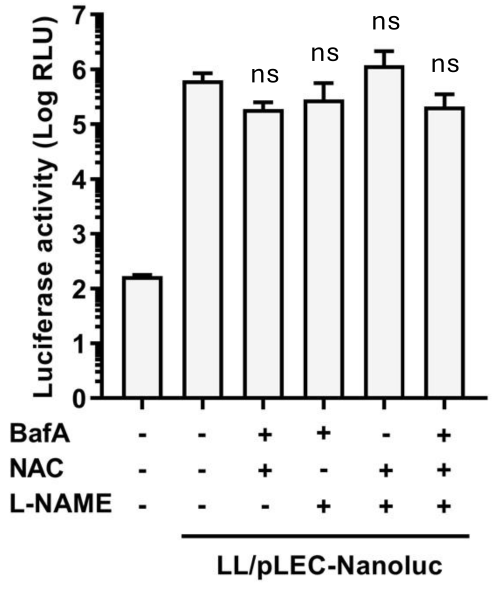

Fig. S10 Effects of inhibitors for bactericidal mechanisms on plasmid transfer from LL to RAW264.7 cells.

(A) The number of viable bacteria in RAW264.7 cells co-cultured with LL/pLEC-Nanoluc (cell-to-bacteria ratio was 1:1,000) for 26 h. Cells were harvested, counted, and lysed with 0.1% Triton x100. Cell lysates were plated on GM17 agar without or with erythromycin (EM - or EM +, respectively). LL colonies were counted after 48 h of incubation. Data for EM + are the same in Fig. 5C. Data are expressed as CFU/cell (mean ± SEM, n=4). nd; not detected, ns; not significant, by Student's t-test. **(B)** Luciferase activity in the cell lysates of RAW264.7 cells co-cultured with LL/pLEC-Nanoluc (cell-to-bacteria ratio was 1:1,000) with the indicated combinations of 40 nM BafA, 2 mM NAC, and 5 mM L-NAME for 20 h. Data are expressed as mean ± SEM. ns; not significant, by one-way ANOVA followed by Dunnett's test (vs. LL/pLEC-Nanoluc with no inhibitor).
